# The role of tumor parenchyma and brain cortex signal intensity ratio in differentiating solitary fibrous tumors and meningiomas

**DOI:** 10.1007/s12672-024-00883-8

**Published:** 2024-02-08

**Authors:** Yue Yu, Fang Gu, Yi-Lin Luo, Shi-Guang Li, Xiao-Feng Jia, Liang-Xian Gu, Guo-Ping Zhang, Xin Liao

**Affiliations:** 1 Department of Radiology, The Affiliated Hospital of Guizhou Medical University, 28 Guiyi Street, Yunyan District, Guiyang, 550004 Guizhou China; 2 Department of Radiology, The Second People’s Hospital of Guiyang, 547 Jinyang South Road, Guanshanhu District, Guiyang, 550023 Guizhou China; 3 Department of Radiology, Dushan County People’s Hospital, No. 1 Ying Shang Road, Baiquan Town, Dushan County, 558299 Guizhou, China

**Keywords:** Diagnosis, Differential diagnosis, Magnetic resonance imaging, Meningioma, Solitary fibrous tumor

## Abstract

**Background:**

Solitary fibrous tumors (SFT) and meningiomas (MA) have similar clinical and radiographic presentations but require different treatment approaches and have different prognoses. This emphasizes the importance of a correct preoperative diagnosis of SFT versus MA.

**Objective:**

In this study, investigated the differences in imaging characteristics between SFT and MA to improve the accuracy of preoperative imaging diagnosis of SFT.

**Methods:**

The clinical and imaging data of 26 patients with SFT and 104 patients with MA who were pathologically diagnosed between August 2017 and December 2022, were retrospectively analyzed. The clinical and imaging differences between SFT and MA, as well as between the various pathological grades of SFT, were analyzed.

**Results:**

Age, gender, cystic change, flow void phenomenon, yin-yang sign, lobulation, narrow base, tumor/cortex signal ratio (TCSR) > 1.0 in T1-weighted imaging (T1WI), TCSR ≥ 1.1 in T2-weighted imaging (T2WI), peritumoral edema, and absence of dural tail sign varied between SFT and MA. As per the receiver operating characteristic (ROC) curve analysis, TCSR > 1 in T1WI has the maximum diagnostic accuracy for SFT. Cranial or venous sinus invasion had a positive effect on SFT (Grade III, World Health Organization (WHO) grading).

**Conclusion:**

Among the many radiological and clinical distinctions between SFT and MA, TCSR ≥ 1 exhibits the highest predictive efficacy for SFT; while cranial or venous sinus invasion may be a predictor of WHO grade III SFT.

## Introduction

Solitary fibrous tumors (SFTs) are a subtype of spindle cell mesenchymal tumor that manifests infrequently within the intracranial space [1, 2]. The term solitary fibrous tumor/hemangiopericytoma was adopted in the 2016 World Health Organization (WHO) classification of neurological tumors [3], which was then revised to SFT in the 2021 WHO classification of neurological tumors [4]. SFT and meningiomas (MA) have similar imaging manifestations, however SFT is more invasive, has rich blood supply, involves severe intraoperative bleeding, and has a higher risk of postoperative dissemination and recurrence [5]; hence, preoperatively distinguishing SFT from MA is crucial for determining the appropriate treatment. In this study, the clinical and imaging data of 26 patients with SFT and 104 patients with MA were compared and analyzed to determine the distinguishing characteristics of SFT and MA.

## Data and methods

### Clinical data

We retrospectively analyzed the clinical and imaging data of 26 patients with intracranial SFT and 104 patients with MA, who were pathologically diagnosed at the Second People’s Hospital of Guiyang City and the Affiliated Hospital of Guizhou Medical University from August 2017 to December 2022. Inclusion criteria: (1) Patients were pathologically determined to have SFT or MA; (2) Patients with MA were matched according to the size and location of SFT and randomly selected in a ratio of 1:4. (3) Patients had complete imaging data and underwent an MRI scan and an MRI enhanced scan. Exclusion criteria: (1) Patients who had undergone surgery or radiotherapy prior to the imagological examination; (2) Image quality did not meet the study requirements for a variety of reasons. This study used the 2016 edition of the World Health Organization (WHO) Classification of Neurological tumors for pathological classification.

### Examination methods

For the examination, Philips Ingenia 3.0 T and GE Sign 3.0 T MRI scanners were utilized. Patients were placed in a supine position and a standard head coil was applied. Parameters of Philips Ingenia 3.0 T scan: T1-weighted imaging (T1WI) repetition time (TR): 425 ms, echo time (TE): 20 ms; T2WI repetition time (TR): 4000 ms, echo time (TE): 100 ms; matrix: 256 × 256 mm, FOV: 240 × 180, thicknesses: 5 mm. During the enhanced scan, a high-pressure syringe was used to inject gadoterate meglumine (Gd-DOTA) through the median cubital vein at a dose of 0.2 mL/kg and a rate of 2 mL/s, and axial, coronal, and sagittal scans were performed with the T1W1 sequence. Parameters of GE Sign 3.0 T scan: T1WI repetition time (TR): 500 ms, echo time (TE): 25 ms; T2WI repetition time (TR): 5000 ms, TE: 106 ms. Matrix: 256 × 512, FOV: 240 × 240 mm, thickness: 5 mm. During the enhanced scan, a high-pressure syringe was used to inject Gd-DOTA through the median cubital vein in bolus at a dose of 0.1 mL/kg and a rate of 2 mL/s, and axial, coronal, and sagittal scans were performed with the T1WI sequence.

### Image analysis

The scan images were uploaded to a picture archiving and communication system (PACS), and were analyzed by two experienced radiologists who specialized in the central nervous system at the PACS workstation, to ensure consensus. The tumor/cortex signal ratio (TCSR) on T1WI and T2WI, internal features of tumor (cystic change, hemorrhage, flow void phenomenon), contour of tumors (lobulate or smooth), the contact way of tumor with the meninges (narrow-base or wide-base), dural tail sign, yin-yang sign, and peritumoral edema were analyzed.

**Fig. 1:**
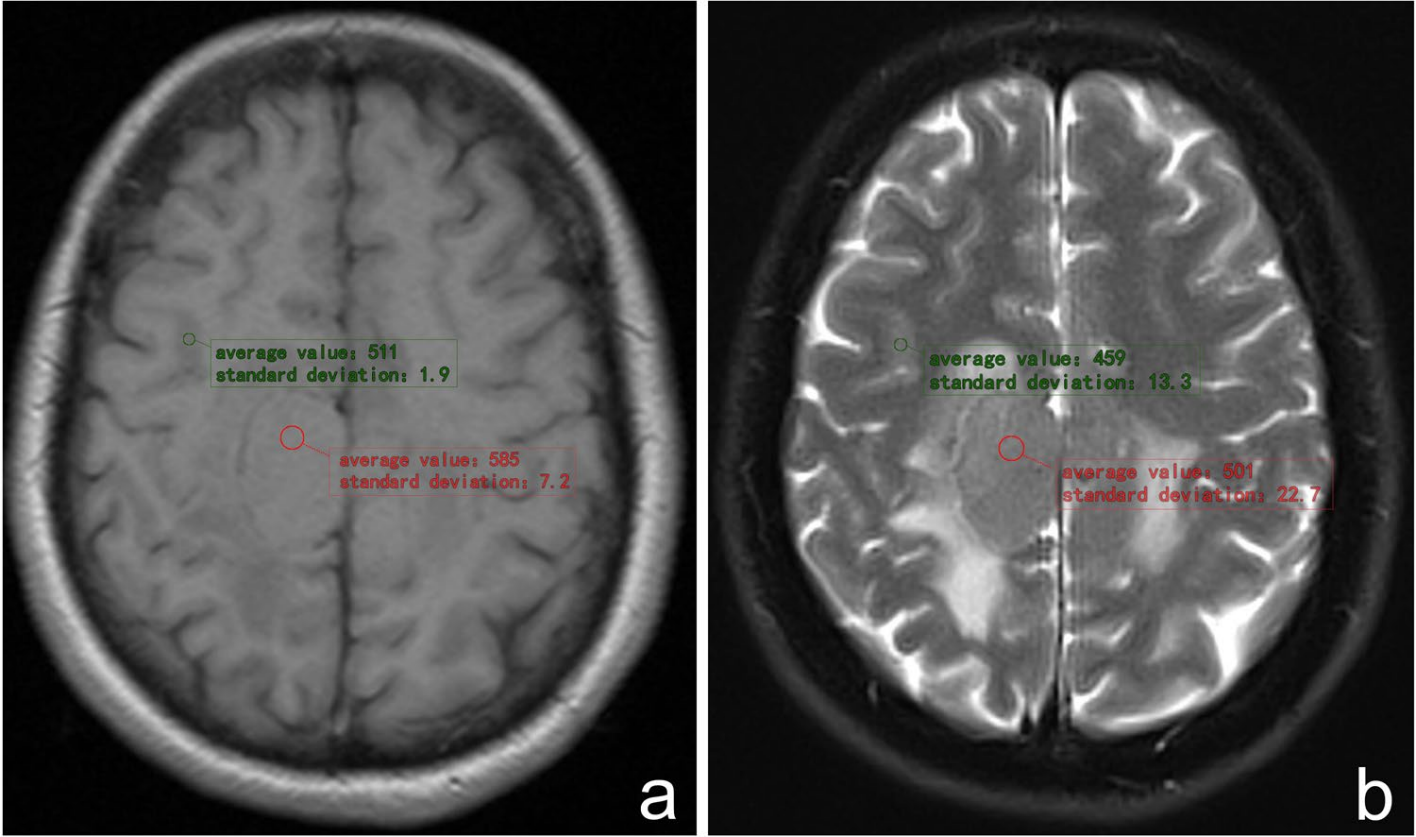
Presentation of TCSR Measurement Method. **a**, **b** This figure shows the placement of regions of interest (ROIs) in T1WI (**a**) and T2WI (**b**) in an SFT case, with annotated average signal intensities for tumor parenchyma (red) and normal cortex (green). Areas are carefully chosen to exclude necrosis, calcification, hemorrhage, and vessels in the tumor, and to avoid sulci or fissures in the cortex. To ensure measurement accuracy, two radiologists independently performed the measurements 3 times, with the average values used to calculate the respective TCSR

TCSR measurement: Tumor parenchyma and normal cortex signal intensities were measured independently, and the ratio between the two was determined. Tumor parenchyma was selected from regions free of necrosis, calcification, hemorrhage, and vessels. If multiple regions with different signal intensities exist within the tumor parenchyma, the area with the highest signal intensity was selected for measurement. For normal cortex, areas without any pathological changes were selected while avoiding regions near the sulci or fissures. Subsequently, the signal intensities of these two regions were measured using tools in the PACS workstation, and TCSR was calculated. To ensure measurement accuracy, this process was repeated 3 times on both T1WI and T2WI MRI images by the two radiologists independently to reduce errors. The final TCSR value was obtained by averaging these six measurements (Fig. 1). The “dural tail sign” refers to a thickening of the dura adjacent to an intracranial pathology on contrast-enhanced T1 MR images [6]. Definition of the “Yin-Yang sign”: On T2WI, tumors have two separate solid components, one of which is hyperintense and the other is iso- to hypointense relative to the brain parenchyma [7].

### Statistical analysis

The available data for the study were generated and entered in MS Excel before being analyzed with SPSS 22.0. The statistical data are expressed as [n (%)], and the chi-squared test was used to compare the two groups; the measurement data were of normal distribution and expressed as mean ± standard deviation, and the *t*-test for independent samples was used to compare the two groups. We utilized the receiver operating characteristic (ROC) curve to evaluate the diagnostic efficacy of clinical and radiological features in distinguishing between SFT and MA. We used a binary logistic regression model to evaluate the impact of intracranial or venous sinus invasion on the occurrence of Grade III SFT. The difference was statistically significant when P < 0.05.

## Results

### Clinical features

In this study, we enrolled a total of 130 cases, including 26 cases of SFT and 104 cases of MA. There were 14 males and 12 females among the 26 patients with SFT, with age ranging from 24 to 76 years and a mean age of 48.19 ± 12.26, and 19 males and 85 females among the 104 patients with MA, with age ranging from 24 to 77 years and a mean age of 56.25 ± 11.24. According to the WHO classification, there was 1 case of grade I, 19 cases of grade II, and 6 cases of grade III among the 26 cases of SFT.

### Pathological results

The 26 cases of SFT manifested as soft tissue tumors that were either gray-white or gray-brown in color. Tumor cells appeared spindle-shaped, intricately intertwined, and arranged irregularly after HE staining. There were cellularly dense regions and fibrotic, cellularly sparse regions, both of which were separated by fibrous stroma. All 22 cases had positive STAT6 immunohistochemistry results (100% positivity rate). Other positive markers included Vim (88%, 23/26), CD99 (96.2%, 25/26), CD34 (81%, 21/26), and Bcl-2 (76.9%, 20/26). Among the 104 cases of MA, the pathological types included transitional type (n = 49), atypical type (n = 17), fibrous type (n = 15), meningothelial type (n = 13), psammomatous type (n = 6), secretory type (n = 1), clear cell variant type (n = 1), metaplastic type (n = 1), and anaplastic type (n = 1).

### Differences in clinical and imaging features across various pathological types of MA

In this study, we analyzed the differences in clinical and imaging features across various pathological types of meningiomas. Due to the limited number of cases, psammomatous type (n = 6), secretory type (n = 1), clear cell variant type (n = 1), metaplastic type (n = 1), and anaplastic type (n = 1) were excluded from the analysis. The statistical results indicated that, apart from cystic change (p = 0.036), there were no significant differences in other clinical and imaging characteristics among the different pathological types of meningiomas (Table 1).

**Table 1:** Comparative analysis of clinical and radiological characteristics across various pathological types of MA

	Meningothelial type (n = 13)	Fibrous type (n = 15)	Transitional type (n = 49)	Atypical type (n = 17)	F	P
Gender	0.27 ± 0.47	0.07 ± 0.26	0.16 ± 0.37	0.29 ± 0.47	1.028	0.384
Age	48.91 ± 6.25	59.07 ± 7.74	56.29 ± 11.47	56.65 ± 14.21	1.887	0.137
Narrow base	0.09 ± 0.30	0.13 ± 0.35	0.08 ± 0.28	0.06 ± 0.24	0.198	0.897
Lobulation	0.36 ± 0.50	0.27 ± 0.46	0.39 ± 0.49	0.65 ± 0.49	1.803	0.152
Cystic change	0.09 ± 0.30	0.20 ± 0.41	0.27 ± 0.45	0.53 ± 0.51	2.979	0.036
Yin-yang sign	0.00 ± 0.00	0.07 ± 0.26	0.06 ± 0.24	0.00 ± 0.00	0.641	0.591
Flow void phenomenon	0.27 ± 0.47	0.33 ± 0.49	0.45 ± 0.50	0.41 ± 0.51	0.397	0.755
Absence of dural tail sign	0.00 ± 0.00	0.20 ± 0.41	0.08 ± 0.28	0.06 ± 0.24	0.758	0.521
Peritumoral edema	0.45 ± 0.52	0.73 ± 0.46	0.76 ± 0.43	0.88 ± 0.33	2.403	0.073
TCSR > 1.0 in T1WI	0.18 ± 0.40	0.20 ± 0.41	0.24 ± 0.43	0.18 ± 0.39	0.231	0.874
TCSR ≥ 1.1 in T2WI	0.91 ± 0.30	0.93 ± 0.26	0.86 ± 0.35	0.76 ± 0.44	0.789	0.503

### Differences in clinical and imaging features of SFT and MA

**Fig. 2:**
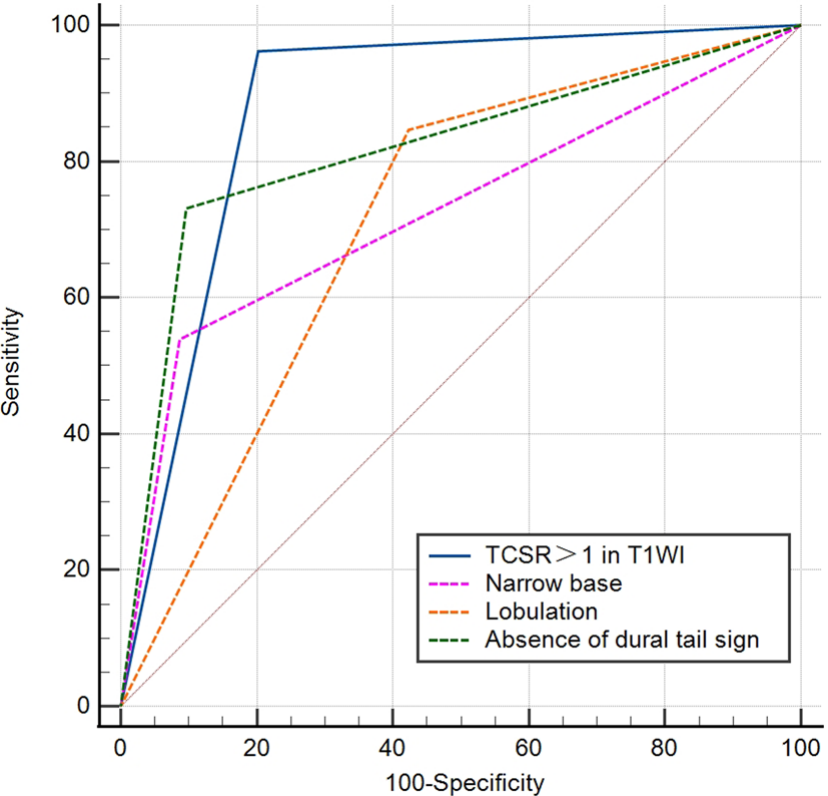
ROC Curve Analysis for Feature Discrimination in SFT vs. Meningioma. The ROC curve highlights the diagnostic accuracy of features differentiating SFT from meningiomas. AUC values: TCSR > 1 in T1WI (0.880), absence of dural tail sign (0.817), narrow base (0.726), and lobulation (0.712)

**Fig. 3:**
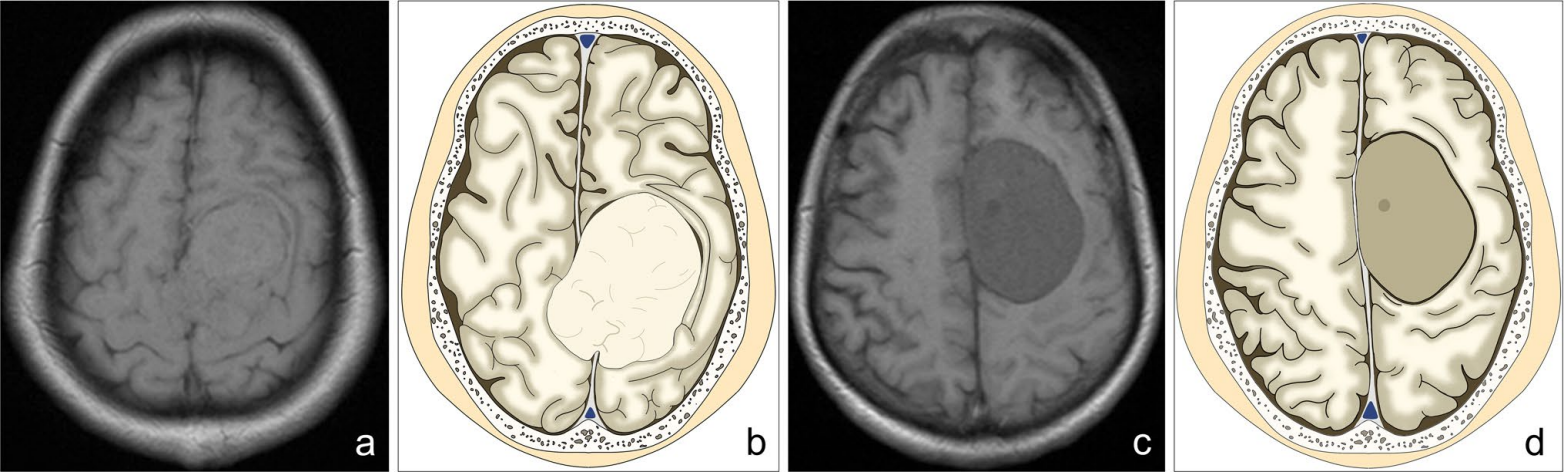
Comparison of tumor parenchymal signal differences in T1WI between SFT and MA. **a**, **b** T1WI of SFT at the cerebral falx and the corresponding mode patterns, with TCSR > 1.0 in T1WI, indicating that the tumor exhibits a slightly higher signal when referenced to the cerebral cortical signal; **c**, **d** T1WI images of MA and the corresponding mode patterns, with TCSR < 1.0 in T1WI, indicating that the tumor exhibits a slightly lower signal when referenced to the cerebral cortical signal, along with the presence of even lower signal cystic changes within the tumor

In addition to statistically significant differences in age, TCSR > 1.0 in T1WI, cystic change, intratumoral hemorrhage, flow void phenomenon, yin-yang sign, lobulation, peritumoral edema, dural tail sign, and narrow base between the two groups, there were more females in the MA group than the SFT group (Table 2). Clinical and radiological features were analyzed using ROC curves to determine their sensitivity, specificity, and area under the curve (AUC) value in distinguishing between SFT and MA (Table 3), and indicators with AUC values between 0.7 and 1 are presented in the ROC (Fig. 2). The sensitivity was lowest for the yin-yang sign, but the specificity was the highest. In contrast, peritumoral edema and TCSR > 1.0 in T1WI displayed the highest sensitivity, with the specificity of peritumoral edema being the lowest. TCSR > 1.0 in T1WI was found to be the most diagnostically effective indicator (Fig. 3).

**Table 2:** Comparative analysis of clinical and imaging features of SFT and MA

	SFT (26)	MA (104)	t/X^2^	P
Age	48.19 ± 12.26	56.25 ± 11.24	− 3.211	0.002
Gender			13.9	< 0.001
Male	14 (53.8%)	19 (18.3%)		
Female	12 (46.2%)	85 (81.7%)		
Slightly high signal of tumor parenchyma in T1WI			62.509	< 0.001
Yes	24 (92.3%)	14 (13.5%)		
No	2 (7.7%)	90 (86.5%)		
TCSR > 1.0 in T1WI			52.493	< 0.001
Yes	25 (96.2%)	21 (20.2%)		
No	1 (3.9%)	83 (79.8%)		
TCSR ≥ 1.1 in T2WI			10.663	0.001
Yes	14 (53.9%)	87 (83.7%)		
No	12 (46.1%)	17 (16.3%)		
Cystic change			11.131	< 0.001
Yes	16 (61.5%)	28 (73.1%)		
No	10 (38.5%)	76 (26.9%)		
Intratumoral hemorrhage			3.538	0.060
Yes	3 (11.5%)	3 (2.9%)		
No	23 (88.5%)	101 (97.1%)		
Flow void phenomenon			5.673	0.017
Yes	17 (65.4%)	41 (39.4%)		
No	9 (34.6%)	63 (60.6%)		
Yin-yang sign			23.08	< 0.001
Yes	10 (38.5%)	5 (4.8%)		
No	16 (61.5%)	99 (95.2%)		
Lobularization			14.896	< 0.001
Yes	22 (84.6%)	44 (42.3%)		
No	4 (15.4%)	60 (57.7%)		
Peritumoral edema			6.020	0.014
Yes	25 (96.2%)	77 (74.0%)		
No	1 (3.8%)	27 (26.0%)		
Absence of dural tail sign			48.334	< 0.001
Yes	19 (73.1%)	10 (9.6%)		
No	7 (26.9%)	94 (90.4%)		
Narrow base			29.172	< 0.001
Yes	14 (53.8%)	9 (8.7%)		
No	12 (46.2%)	95 (91.3%)		
Invaded bone or venous sinus			2.783	0.095
Yes	7 (26.9%)	14 (13.5%)		
No	19 (73.1%)	90 (86.5%)

**Table 3:** Single Imaging or Clinical Markers for Discriminating SFT from MA: AUC, Sensitivity, and Specificity Analysis

Features	AUC	Sensitivity	Specificity	95% CI
Age	0.699	0.644	0.769	0.585–0.813
Gender	0.678	0.538	0.817	0.554–0.802
Narrow base	0.726	0.538	0.913	0.602–0.850
Lobulation	0.712	0.846	0.577	0.608–0.815
Cystic change	0.673	0.615	0.731	0.553–0.793
Yin-yang sign	0.668	0.385	0.952	0.537–0.800
Flow void phenomenon	0.630	0.654	0.606	0.511–0.749
Absence of dural tail sign	0.817	0.731	0.904	0.711–0.924
Peritumoral edema	0.611	0.962	0.260	0.502–0.720
TCSR > 1 in T1WI	0.880	0.962	0.798	0.814–0.945
TCSR ≥ 1.1 in T2WI	0.638	0.846	0.462	0.522–0.776

### Comparison of clinical and imaging features of SFTs with grade II and grade III in the WHO classification

**Fig. 4:**
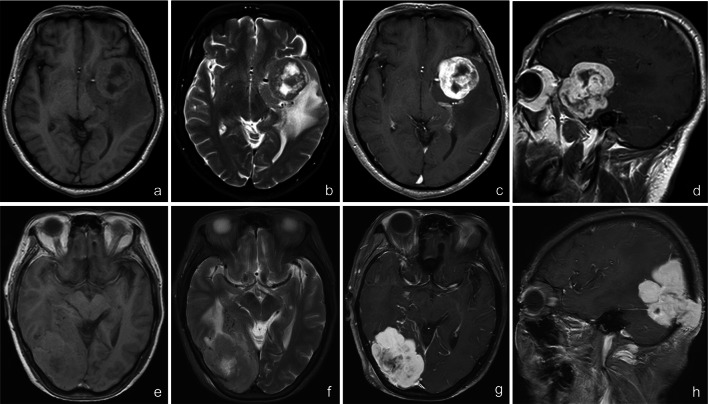
Imaging characteristics of WHO-classified grade II and grade III SFTs. (a-d) Male, 52 years old, diagnosed with grade II SFT at the left sphenoid ridge; **e**–**h** female, 76 years old, diagnosed with grade III SFT at the right occipitalia; **a**, **b** axial T1WI and T2WI showed (using cerebral cortex signal as the reference) slightly high signals of tumor parenchyma in T1WI (TCSR > 1.0 in T1WI), slightly low signals of tumor parenchyma in T2WI (TCSR < 1.0 in T2WI), with cystic change area within the lesions and edema zone surrounding the lesions; **c**, **d** axial and sagittal T1WI enhancement demonstrated uneven enhancement of masses and no enhancement of the cystic change area. **e**, **f** axial T1WI and T2WI showed (using cerebral cortex signal as the reference) that the tumor parenchyma had mixed equal and slightly high signal in T1WI (TCSR > 1.0 in T1WI) and iso-signal in T2WI (TCSR = 1.0 in T2WI)—the cystic area was seen within the lesion, and edema zone was seen around the lesion; **g**, **h** axial and sagittal T1WI enhancement showed that masses were unevenly and obviously enhanced, the cystic change area was not enhanced, and the adjacent bone tissue was invaded

The group with grade II was compared with the group with grade III, and the two groups differed statistically only in cranial or venous sinus invasion (Table 4). Additional analysis by binary logistic regression revealed that cranial or venous sinus invasion has a significant positive effect on SFT with grade III, *P* = 0.027, OR = 10.667, 95% CI (1.309, 86.933) (Fig. 4).

**Table 4:** Comparative analysis of clinical and imaging features of grade II and grade III SFTs as per the WHO grading

	SFT (n = 19) with grade II	SFT (n = 6) with grade III	t/X2	P
Age	49.47 ± 10.00	43.83 ± 19.02	0.961	0.346
Gender			0.013	0.91
Male	10 (52.6%)	3 (50%)		
Female	9 (47.4%)	3 (50%)		
Slightly high signal of tumor parenchyma in T1WI				1.000
Yes	18 (94.7%)	6 (100%)		
No	1 (5.3%)	0 (0%)		
TCSR ≥ 1.2 in T1WI			1.176	0.278
Yes	5 (26.3%)	3 (50.0%)		
No	14 (73.7%)	3 (50.0%)		
TCSR ≥ 1.2 in T2WI			0.680	0.409
Yes	9 (47.37)	4 (66.7%)		
No	10 (52.63)	2 (33.3%)		
Cystic change			1.281	0.258
Yes	11 (57.9%)	5 (83.3%)		
No	8 (42.1%)	1 (16.7%)		
Intratumoral hemorrhage			0.806	0.369
Yes	1 (5.3%)	1 (16.7%)		
No	18 (94.7%)	5 (83.3%)		
Flow void phenomenon			0.672	0.412
Yes	13 (68.4%)	3 (50%)		
No	6 (31.6%)	3 (50%)		
Yin-yang sign			1.791	0.181
Yes	9 (47.4%)	1 (16.7%)		
No	10 (52.6%)	5 (83.3%)		
Lobularization			0.003	0.959
Yes	16 (84.2%)	5 (83.3%)		
No	3 (15.8%)	1 (16.7%)		
Peritumoral edema				1.000
Yes	18 (94.7%)	6 (100%)		
No	1 (5.3%)	0 (0%)		
Absence of dural tail sign			0.111	0.739
Yes	5 (26.3%)	2 (33.3%)		
No	14 (73.7%)	4 (66.7%)		
Narrow base			1.102	0.294
Yes	8 (42.1%)	4 (66.7%)		
No	11 (57.9%)	2 (33.3%)		
Invaded bone or venous sinus			5.855	0.016
Yes	3 (15.8)	4 (66.7)		
No	16 (84.2)	2 (33.3)

## Discussion

Intracranial SFT is extremely uncommon, accounting for only 0.09% of intracranial meningeal-associated tumors [8] and occurring more frequently in adults, with a similar incidence in males and females. The lesions can be found in meningeal distribution areas including the tentorium, convex surface of brain, falx cerebri, and skull base [9]. The clinical manifestations are nonspecific, with headache being the most prevalent symptom [10]. There is some overlap in the clinical and imaging features and lesion sites between intracranial SFT and MA, although both conditions are treated and prognosed very differently. Aggressive therapeutic interventions such as surgery and subsequent radiochemotherapy are often necessary after a diagnosis of SFT has been made. When dealing with MA, however, a more conservative strategy may be appropriate. Therefore, it is crucially important to correctly differentiate between SFT and MA prior to surgery [11]. Several imaging differences have been proposed to differentiate SFT from MA, but SFT is still frequently misdiagnosed. In this study, we compared multiple clinical and imaging features of SFT and MA to show for the first time that there is a subtle difference in T1WI tumor parenchymal signals between SFT and MA, and that this difference is highly effective in the differential diagnosis of SFT and MA; this finding has the potential to provide a new, more direct approach to the diagnosis and differential diagnosis of SFT.

We observed that SFT is more frequently associated with TCSR > 1 on T1WI, while MA is often found with TCSR ≤ 1 on T1WI. This suggests that, using cerebral cortical signal as a reference, the majority of SFT parenchyma exhibit slightly high signal in T1WI, whereas the majority of MA parenchyma exhibit iso-signal or slightly low signal. This may be due to the difference in relaxation time in T1 caused by the different water content of the two tumors, with the water content of SFT tumor parenchyma being lower. Xiao et al. also found that a higher proportion of SFT tumors exhibited an iso-/high- signal in T1WI [12]. Using the white matter signal as a reference, He et al. discovered that the SFT and MA signals in T1WI and T2WI differed from one another [13]. There are some studies that suggest that there is no difference between SFT and MA signals in T1WI [14], however, we noticed that the evaluation of high, moderate, or low tumor signal in these studies did not specify the corresponding reference standard, so it is difficult to rule out the possibility that the authors subjectively determined the grading of tumor signal value. In fact, the T1WI images of SFT presented by the authors of some published works are annotated as having low signal [15, 16], whereas our reference standard dictates that the signal should be slightly high i.e., TCSR > 1 in T1WI.

**Fig. 5:**
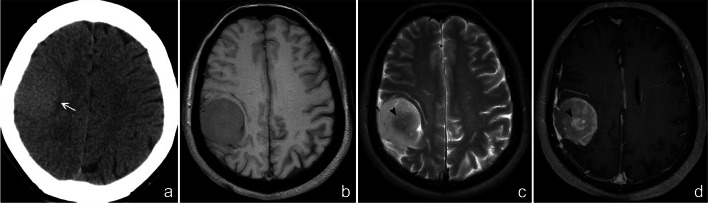
Imaging features of the “yin-yang sign” in MA. **a**–**d** CT scan and MRI images of MA at the convexity of the right parietal brain. **a** head CT scan of tumor parenchyma showed iso-intensity with no calcification in the lesion (arrow); **b** signal of tumor parenchyma in T1WI showed iso-signal (using cerebral cortex signal as the reference); **c**, **d** T2WI and T1WI enhancement showed that areas within the tumor having low signals in the T2WI were more significantly enhanced in the T1WI (black arrow), also known as the “yin-yang sign”

The results of this study demonstrated a different gender distribution between MA and SFT—a female predominance in the MA group, and younger patients in the SFT group. Intra-tumor cystic changes, hemorrhage, flow void phenomenon, yin-yang sign, lobulated contour, narrow base, absence of dural tail sign, and peritumor edema were more prevalent in SFT compared to MA, similar to the results of previous studies [12, 13, 16–19]. Among these imaging features, the yin-yang sign was interpreted as low signals in the collagen fibrous tissue-rich areas of the tumors in T2WI and high signals in the dense portion of the tumor cells, with more significant enhancement in the low signal areas after enhancement; therefore, the sign is considered a specific imaging feature of SFT [20, 21] and was observed in 9 (47.4%) cases of SFT in the present study; however, for the first time, (Fig. 5) it was also found that the “yin-yang sign” was also present in five cases of MA (4.8%), suggesting that it is not exclusive to SFTs.

SFT is categorized into 3 grades—grade I SFTs are benign tumors for which resection is the primary treatment, while grade II and III SFTs are moderately invasive and considered malignant, requiring radiation therapy to prevent recurrence and dissemination along with resection [22]. Compared to SFT of grade II, SFT of grade III is more invasive and is associated with higher disease-related mortality and morbidity [23]. Imaging findings suggest a tendency for grade III SFTs to show more instances of bone destruction or venous sinus invasion compared to grade II SFTs, whereas there were no differences in signal features, morphology, peritumoral edema, or dural tail signs between the lesions of the two grades. However, given the limited number of grade III SFT cases, further research for validation in the future is required based on these observational results.

This study has certain limitations. Due to the low incidence of solitary fibrous tumors (SFT), the sample size was relatively limited, especially for grade III SFT cases, which may affect the generalizability of the study results. Additionally, as a retrospective analysis, this study might be subject to some degree of selection bias. Future research will consider increasing the sample size and conducting multi-center collaborations to enhance the applicability of the study findings.

## Conclusion

Multiple clinical and radiological differences between SFT and MA were identified in the present study, which are useful in improving the precision of preoperative differential diagnosis. The significance of TCSR > 1.0 in T1WI was the most striking and novel finding; this indicated superior diagnostic efficacy for SFT compared to other parameters. Additionally, WHO grade III SFT was found to have a higher likelihood of cranial or venous sinus invasion, providing further insights into prognostic stratification within SFT.

## Data Availability

The datasets used and analysed during the current study available from the corresponding author on reasonable request.

## Abbreviations

SFT: Solitary Fibrous Tumor
MA: Meningiomas
TCSR: The Tumor/Cortex Signal Ratio
T1WI: T1-weighted imaging
T2WI: T2-weighted imaging
ROC: Receiver operating characteristic
AUC: Area under the curve
WHO: World Health Organization
MRI: Magnetic resonance imaging
TR: Repetition time
TE: Echo time
FOV: Field of view
Gd-DOTA: Gadoterate meglumine
PACS: Picture archiving and communication system
SPSS: Statistical package for the social sciences
